# Dengue Virus Biosafety: An Analysis of Evidence, Global Inconsistencies, and Risk Gaps

**DOI:** 10.1089/apb.2024.0065

**Published:** 2025-08-29

**Authors:** Khanh Kim Le, Stuart D. Blacksell

**Affiliations:** ^1^ Mahidol-Oxford Tropical Research Medicine Unit, Faculty of Tropical Medicine, Mahidol University, Bangkok, Thailand.; ^2^ Centre for Tropical Medicine and Global Health, Nuffield Department of Medicine, Department of Medicine Research Building, University of Oxford, Nuffield, Oxford, United Kingdom.

**Keywords:** dengue virus, DENV, biosafety, biosecurity, global biosafety standards

## Abstract

**Introduction:**

Dengue virus (DENV) poses a significant global health threat, particularly in tropical and subtropical regions, where it is primarily transmitted by *Aedes* spp. mosquitoes. Its biosafety and biosecurity management present unique challenges due to both its vector-borne nature and rare instances of nonvector transmission.

**Methods:**

This study reviews existing practices in laboratory containment, risk group classifications, and infectious dose data related to DENV. We conducted a global analysis of current biosafety standards, identifying gaps and inconsistencies in containment protocols and risk group designations across various regions.

**Discussion and Conclusion:**

Our review reveals substantial variability in global standards for biosafety levels and risk group classifications. These inconsistencies complicate DENV research, containment efforts, and responses to outbreaks. The lack of harmonization between different countries’ guidelines has hindered efforts to mitigate risks associated with DENV handling and outbreak management. The findings underscore the urgent need for harmonized, evidence-based guidelines to standardize biosafety practices globally. A unified approach to laboratory containment, risk group classifications, and infectious dose data will help mitigate risks, improve containment, and enhance preparedness for DENV outbreaks.

**Significance:**

Addressing these biosafety and biosecurity inconsistencies is crucial for strengthening global health security. Improved standards and consistent practices will reduce the burden of DENV-related diseases and enhance global preparedness to manage future outbreaks effectively.

## Introduction

The World Health Organization (WHO) released the fourth edition of the Laboratory Biosafety Manual (LBM4) in 2020,^1^ which advocates for risk-based biosafety using established knowledge. The WHO LBM4 emphasizes risk-based biosafety to improve laboratory biological risk management, particularly in low-resource settings.^2^ This review discusses the general characteristics, biosafety evidence, and other important information regarding dengue virus (DENV). Our review also highlights gaps in the current evidence and regulatory inconsistencies. It provides recommendations for sustainable risk-based biosafety practices while working with DENV using the principles promoted by the WHO.

### General Characteristics

DENV belongs to the *Flaviviridae* family and the Flavivirus genus and is characterized by its enveloped, single-stranded RNA genome.^3^ Genetically, DENV is classified into four distinct serotypes—DENV-1, DENV-2, DENV-3, and DENV-4, based on antigenic differences in the E envelope protein.^3^ These serotypes share approximately 65–70% nucleotide homology, but each can provoke specific immune responses upon infection.^4^

#### Clinical disease

DENV infection can range from mild, flu-like symptoms to severe illness, encompassing three phases: febrile, critical, and convalescent. The clinical presentation typically begins with a sudden onset of fever, severe headache, pain behind the eyes, joint and muscle pain, rash, and mild bleeding (such as nose or gum bleed). This acute febrile phase lasts 2–7 days.^5^ Severe dengue forms typically occur during the critical phase of the disease. In some cases, the disease progresses to severe dengue, previously known as dengue hemorrhagic fever or dengue shock syndrome.^6^ These severe symptoms are characterized by plasma leakage, fluid accumulation, severe bleeding, and organ impairment, which can lead to shock and death if not managed promptly. The convalescent phase, also known as the recovery phase, occurs after the critical phase of dengue infection and typically lasts 2–3 days. This phase is marked by significant improvement and recovery from the acute symptoms of dengue.^5^ Therefore, early diagnosis and proper clinical management are critical to reducing mortality rates associated with severe dengue.

#### Modes of transmission

DENV is primarily transmitted through the bite of infected *Aedes* spp. mosquitoes, with *Aedes aegypti* and *Aedes albopictus* being the main vectors. Other *Aedes* spp. can also serve as less common vectors, such as *A. polynesiensis*,^7^ *A. scutellaris*,^8^ and *A. hensilli*.^9^ In addition to vector-borne transmission, different modes of DENV transmission have been identified. Vertical transmission, although less common, can occur when an infected pregnant woman passes the virus to the fetus, which can lead to neonatal dengue.^10–15^ Rare cases of nonvector transmission have also been reported, including transmission through blood transfusions^16,17^ and organ transplants,^18–20^ which underscore the importance of screening blood and organ donations for DENV, especially in endemic areas. Occupationally acquired DENV infections in laboratory and healthcare settings (covered in detail in a later section). These infections may arise from exposures to DENV-infected patient samples or animals through aerosol or parenteral routes,^21–23^ including needlestick injuries^24–26^ and DENV-infected mosquitoes.^27–29^

#### Treatment and prophylaxis

There is no specific antiviral treatment for dengue infection; management primarily relies on preventive measures and supportive care. Dengue is transmitted by *Aedes* mosquitoes, making mosquito control measures crucial in preventing infection. Acetaminophen (paracetamol) is commonly employed to reduce fever and alleviate pain, but nonsteroidal anti-inflammatory drugs should be avoided due to the risk of bleeding. Dengue vaccination, such as Dengvaxia, is available in certain countries and is recommended for individuals with a prior history of dengue infection in regions with a high dengue burden. However, the complications observed in the Philippines are related to antibody-dependent enhancement (ADE), where prior vaccination in seronegative individuals led to more severe disease upon subsequent infection.

Furthermore, Dengvaxia exhibits the highest efficacy against the DENV-4 serotype and the lowest against DENV-2, although the underlying reasons remain unclear. The vaccine’s uptake has also been limited due to the requirement of three doses administered 6 months apart. The WHO recommends Dengvaxia for individuals with a confirmed previous dengue infection due to the risk of ADE in seronegative individuals. In August 2022, the Indonesian Food and Drug Administration approved the Qdenga vaccine to protect against all four DENV serotypes in individuals aged 6–45 years.^30^ Other vaccines and treatments are under development, aiming to provide broader protection and address the global burden of dengue, which affects millions of people annually, particularly in tropical and subtropical regions.^31^

### Laboratory Biosafety

#### Risk group classification and biosafety levels

The classification of the DENV varies internationally (Table 1). In the United States, the *Biosafety in Microbiological and Biomedical Laboratories* (*BMBL*) 6th edition, Australia/New Zealand Standard AS/NZS 2243.3:2010, Canada (2023), and National Institutes of Health (NIH) guidelines classify DENV as a risk group 2 (RG2) pathogen. Conversely, standards from Switzerland (2013), Belgium (2008), the European Union Directive 2000/54/EC (2020), and the U.K. Advisory Committee on Dangerous Pathogens (ACDP) classify it as a risk group 3 (RG3) pathogen. This difference emphasizes the variability in the risk assessment and classification of DENV worldwide.

**Table 1.** Summary of risk group designation for dengue virus based on the ABSA International database

Guidance or standard	Risk group	Reference
United States *Biosafety in Microbiological and Biomedical Laboratories*, 6th edition	2	^32^
Australia/New Zealand Standard AS/NZS 2243.3:2010	2	^33^
Canada (2023)	2	^34^
National Institutes of Health (NIH) guidelines	2	^35^
Singapore (2023)	3	^36^
Belgium (2008)	3	^37^
European Union Directive 2000/54/EC (2020)	3	^38^
United Kingdom Advisory Committee on Dangerous Pathogens (ACDP)	3	^39^

#### Infectious dose

The infectious dose of DENV in humans remains poorly characterized, and a summary of available evidence is presented in Table 2. Existing literature indicates that DENV is highly contagious, with fewer than 10 plaque-forming units (PFU) capable of causing infection.^34^ Human plasma viremia levels during DENV infection ranged from 10^5^ to 10^8^ copies/mL.^44^ In addition, the doses used to transmit DENV to mosquitoes are primarily for research and vaccine development in the laboratory. The virus concentrations in blood meals provided to mosquitoes ranged from 10^3.74^ to 10^5.74^ PFU/mL, with a median concentration of 10^4.74^ PFU/mL.^41^ F1 generation mosquitoes fed blood meals containing sylvatic or epidemic DENV strains at concentrations of 1.6 × 10^7^ 50% Tissue Culture Infectious Doses/mL (TCID_50_/mL) or 10^6.5^ TCID_50_/mL demonstrated low susceptibility to infection, indicating that these mosquitoes did not readily become infected or support substantial viral replication at these concentrations.^42^ The 50% infectious dose (MID_50_) for *A. aegypti* varied by DENV serotype: MID_50_ values for DENV-1 and DENV-2 were 6.51 ± 0.33 log10-copies/mL and 6.29 ± 0.23 log10-copies/mL, respectively. In contrast, the MID_50_ values for DENV-3 and DENV-4 were approximately 10-fold higher, suggesting that a higher viral concentration is required to infect 50% of mosquitoes.^43^

**Table 2.** Evidence for dengue virus infectious dose in humans and mosquitoes

Year	Transmission	Evidence	Reference
2014	Human	Human ID is <10 PFU. Fewer than 10 PFU led to infection in 50% of volunteers treated with an attenuated dengue virus vaccine candidate.	^34^
2019	Human	Inbred wild-type A. aegypti mosquitoes were orally challenged with two infectious doses (10^8^ and 10^5^ DENV copies/mL), representative of plasma viremia ranges in humans.	^40^
2014	Mosquito	Among isolates, infectious doses were distributed from 10^3.74^ to 10^5.74^ PFU/mL in each blood meal (median 10^4.74^ PFU/mL).	^41^
2008	Mosquito	A low susceptibility to both sylvatic and epidemic DENV-2 virus strains was obtained after ingestion of blood meals with a virus titer of 1.6 × 10^7^ TCID_50_/mL and/or 10^6.5^ TCID_50_/mL.	^42^
2013	Mosquito	The estimated log_10_ MID_50_ [±95% confidence interval (CI)] was similar for DENV-1 (6.51 ± 0.33 log_10_-copies/mL) and DENV-2 (6.29 ± 0.23 log_10_-copies/mL) and ∼10 times higher for DENV-3 (7.52 ± 0.59 log_10_-copies/mL) and DENV-4 (7.49 ± 0.63 log_10_-copies/mL).	^43^

PFU - Plaque forming unit; DENV - Dengue virus; TCID50 - 50% Tissue culture infectious doses.

#### Biocontainment and personal protective equipment

Handling DENV in laboratory settings requires strict adherence to biocontainment and personal protective equipment (PPE) protocols to ensure safety and prevent contamination. A summary of global guidance or standards is presented in Table 3. DENV is typically handled under Biosafety Level 2 (BSL-2) conditions, as recommended by the guidance or standards, including the U.S. *BMBL* 6th Edition,^32^ Australian and New Zealand AS/NZS 2243.3:2010,^33^ Canadian Pathogen safety data sheets,^34^ and U.S. NIH Guidelines^35^ In situations involving high concentrations of the virus or procedures that could increase the risk of exposure, BSL-3 protocols may be necessary, with requirements according to *BMBL* 6th edition,^32^ European Union Directive 2000/54/EC (2020),^38^ and ACDP, UK.^39^ These include stringent controls such as a controlled airflow system directing air from clean to contaminated areas, double-door entry with showers, and HEPA-filtered exhaust systems containing potential contaminants. When working with DENV at BSL-2, PPE includes laboratory coats or gowns, gloves, and eye protection. At BSL-3, more stringent PPE is recommended including facial protection consisting of face shields or safety goggles to protect against infectious splashes, along with N95 respirators or equivalent masks to minimize the risk of exposure to aerosols and full-body protective clothing.

**Table 3.** Summary of global biosafety and biocontainment requirements for working with DENV in a laboratory setting

Country	Regulation	Recommended containment requirements/ Biosafety level (BSL) (low-risk activities)	Recommended containment requirements/ BSL (high-risk activities)
United States	Biosafety in Microbiological and Biomedical Laboratories 6th edition^32^	BSL-2	“Situations may arise for which enhancements to BSL-3 practices and equipment are required; for example, when a BSL-3 laboratory performs diagnostic testing on specimens from patients with hemorrhagic fevers thought to be due to dengue or yellow fever viruses. When the origin of these specimens is Africa, the Middle East, or South America, such specimens might contain etiologic agents, such as arenaviruses, filoviruses, or other viruses that are usually manipulated in a BSL-4 laboratory. Examples of enhancements to BSL-3 laboratories include: 1) enhanced respiratory protection of personnel against aerosols; 2) HEPA filtration of exhaust air from the laboratory; and 3) personal body shower upon exit. Additional appropriate training is recommended for all staff, including animal care personnel.”
Australia	AS/NZS 2243.3:2010^33^	Physical containment level 2 (PC-2) This level of laboratory or facility with its practices and equipment is applicable to research, diagnostic, and other premises where work is carried out with microorganisms or material likely to contain microorganisms that are classified as Risk Group 2 microorganisms. If working with specimens containing microorganisms transmissible by the respiratory route or if the work produces a significant risk to humans or the environment from the production of infectious aerosols, a biological safety cabinet shall be used.	
Canada	Pathogen safety Data Sheets (2023)^34^	“Containment Level 2 facilities, equipment, and operational practices for work involving infectious or potentially infectious materials, animals, or cultures. Lab coat. Gloves when direct skin contact with infected materials or animals is unavoidable. Eye protection must be used where there is a known or potential risk of exposure to splashes. All procedures that may produce aerosols, or involve high concentrations or large volumes should be conducted in a biological safety cabinet (BSC). The use of needles, syringes, and other sharp objects should be strictly limited. Additional precautions should be considered with work involving animals or large scale activities”	
United States	NIH guidelines^35^	“Careful consideration should be given to the types of manipulation planned for some higher Risk Group agents. For example, the RG2 dengue viruses may be cultured under the Biosafety Level (BL) 2 containment (see Section II-B); however, when such agents are used for animal inoculation or transmission studies, a higher containment level is recommended”	
European Union	European Union Directive 2000/54/EC (2020)^38^		Containment level 3
United Kingdom	U.K. Advisory Committee on Dangerous Pathogens^39^		Containment level 3

Insectaries housing *A. aegypti* mosquitoes infected with DENV require stringent biocontainment measures to prevent accidental escape and ensure researcher safety. These facilities should operate under Arthropod Containment Level 2 (summarized in Table 4) or higher, as recommended by the American Committee of Medical Entomology (ACME).^45,46^ Similar guidance is provided for U.K.-specific legislation including the Control of Substances Hazardous to Health, the Specified Animal Pathogens Order,^47^ and European legislation (e.g., quarantine species under EU Regulation 2016/2031).^48^ Detailed risk assessments, facility design tailored to containment needs, and regular monitoring ensure environmental and personnel safety.^45,46,49^ Key requirements include secure double-door entry systems, High Efficiency Particulate Air (HEPA) filtration for air exhaust, and finely meshed screens on windows to prevent mosquito egress. Infected mosquitoes must be fed in gloveboxes to protect personnel from accidental bites, as documented cases of laboratory-acquired DENV infections highlight this risk. When working with mosquitoes infected with the DENV, appropriate PPE is essential to minimize the risk of accidental infection. Full-body protection, such as disposable Tyvek coveralls, is recommended to prevent mosquito bites and contamination, with tightly woven insect-repellent-treated clothing as an alternative if coveralls are unavailable. Double-layer nitrile or latex gloves should be worn, particularly during high-risk activities such as feeding or sorting mosquitoes. Shoe or boot covers are necessary to prevent the transfer of infectious material or mosquito eggs outside the containment area, and head coverings or hoods ensure that mosquitoes do not cling to hair. Gloveboxes should be used for blood-feeding, providing an additional physical barrier. Electric zappers or mosquito-killing devices should be on hand to immediately neutralize escapees. All PPE must be donned appropriately and doffed according to biosafety protocols, with contaminated materials disposed of in designated biohazard containers. Regular training and adherence to these protective measures are critical to maintaining safety.

**Table 4.** Summary of Arthropod Containment Level 2 requirements for working with dengue virus-infected mosquitoes based on the American Committee of Medical Entomology recommendations^44,53^

Purpose of ACL-2	Designed to contain arthropods, including mosquitoes, that may be infected with pathogens of low-to-moderate risk (e.g., hazard group 2 pathogens), to protect personnel, prevent escape, and avoid pathogen dissemination.
Physical facility requirements	- Double-door entry systems (recommended to prevent escape). - Sealed windows or use of fine mesh for containment. - Smooth, light-colored walls and floors for easy detection and cleaning. - Ventilation systems with insect-proof mesh covers to prevent escape through ducts.
Environmental controls	- Maintain specific temperature (e.g., 27 ± 3°C for *Aedes aegypti* colonies). - Humidity levels of 70%–80% for adult mosquito survival. - Controlled photoperiods (e.g., 12:12 or 14:10 h light-dark cycles). - Systems to monitor temperature and humidity.
Operational practices	- Restricted access to trained personnel only. - Daily monitoring and recording of environmental conditions. - Clear marking of restricted areas. - Use of personal protective equipment (PPE), such as gloves and laboratory coats.
Risk mitigation measures	- Nonhand operated sinks with fine mesh drain covers to prevent escape of larvae/eggs. - Decontamination facilities at exits. - Use of insecticides/pesticides in drains as a last resort. - Secure containers for all life stages of mosquitoes (e.g., larval trays, adult cages). - Waste disposal protocols for biological material (e.g., larvae, pupae, adults).
Handling of mosquitoes	- Use of specialized cages with fine mesh and long-sleeved openings to prevent escape. - Handling eggs, larvae, and pupae with precision tools (e.g., Pasteur pipettes, suction tubes). - Transport and storage of eggs under controlled conditions to ensure species integrity.
Containment breach Protocols	- Airlocks or secondary barriers (curtains, safety vestibules). - Emergency response plans, including use of insecticides and sealing escape routes. - Visual inspections of PPE for potential escapees before exiting.

#### Laboratory or healthcare-acquired infections

At least nine documented cases of laboratory-acquired DENV infections have been reported across various countries over the past five decades, underscoring critical gaps in biosafety practices and infrastructure (summarized in Table 5). The documented causes of these infections fall into three primary categories: non-needlestick DENV aerosol or non-needlestick parenteral exposure (*n* = 3), accidental needlestick injuries (*n* = 3), and mosquito bites from infected vectors (*n* = 3). One of the earliest cases occurred in Nigeria (1968), where a laboratory assistant contracted dengue after handling mice inoculated with field samples and washing their cages without proper PPE.^23^ Another significant case in India (1983) involved a laboratorian bitten by a DENV-infected mosquito that escaped during experiments involving sera from the 1982 Delhi dengue epidemic, likely resulting from inadequate containment measures for experimental mosquitoes. In Taiwan (2004), poor biocontainment design in a mosquito research lab led to the escape of infected mosquitoes, resulting in a graduate student contracting DENV-1.^29^ In South Korea (2014), a technician in a BSL-2 facility accidentally pricked herself with a needle while transferring DENV-2 virus solution for filtration.^24^ Similarly, in the United States (2018), a researcher working with DENV-4 experienced frequent splashes during experiments, failed to change gloves consistently, and inadequately washed their hands,^21^ compounded by a small wound on their finger, which likely became contaminated. These incidents illustrate the multifaceted risks associated with laboratory work involving DENV, ranging from direct contact with contaminated materials to indirect exposure via inadequate facilities or procedural errors. Investigations in DENV insectaries with laboratory-acquired infections (LAIs) revealed structural deficiencies, including the absence of double-screened doors, which facilitated mosquito escapes and posed risks to personnel. They collectively highlight the need for robust biosafety protocols, consistent use of PPE, improved laboratory design to contain infected mosquitoes, and comprehensive training for personnel handling infectious agents.

**Table 5.** Evidence describing laboratory or hospital-acquired DENV infections

Year	Country	Occupations	Evidence	Cause	Reference
1968	Nigeria	Laboratory technologist, entomologist, laboratory assistant	A laboratory assistant contracted dengue fever after handling mice inoculated with field samples and washing mouse cages. He experienced a sudden fever and developed a rash on his trunk 4 days later.	Possible DENV aerosol or parenteral exposure	^23^
1980	Japan	Research laboratorian	The researcher was inoculated with a human sample known to contain two types of dengue virus.	Needlestick injury	^26^
1983	India	Laboratorian	Laboratorian received a bite from an infected mosquito that had been inoculated with sera specimens collected during the 1982 Delhi dengue epidemic. One mosquito escaped and bit the laboratorian. The bite was noticed and the mosquito was killed.	Bite from DENV-infected mosquito	^28^
1992	Japan	Nurse	A Japanese female in her thirties, a nurse in Tokyo, was admitted to the hospital in January 1992, 3 days after the onset of fever, headache, and general malaise. Five days before the onset of her symptoms, she had pricked her finger with an injection needle used to draw blood from a febrile patient infected with DENV. She was diagnosed with DENV infection based on three findings: detection of the DENV genome in serum, isolation of DENV from serum, and serum samples were positive for DENV IgM antibodies.	Needlestick injury	^25^
2002	United States	Healthcare worker	While transferring blood from a syringe to a blood culture bottle, the needle dislodged, causing blood to splash onto patient’s face, including her eye, nose, and mouth.	Possible DENV aerosol or parenteral exposure	^21^
2004	Taiwan	Graduate student	A graduate student in central Taiwan researching antibacterial genes in mosquitoes contracted local type 1 dengue fever. The Taiwan CDC found that the laboratory lacked proper design to separate infected and healthy mosquitoes, and the entrance lacked double screening, allowing infected mosquitoes to escape and potentially infect personnel.	Bite from DENV-infected mosquito	^29^
2011	Australia	Scientist	Ten days before hospitalization, they conducted a routine experiment infecting a colony of mosquitoes with DENV-2. Despite wearing appropriate PPE, they reported being bitten by a nonblood fed mosquito that had escaped. They denied any needlestick injury or contact with the blood/virus mixture.	Bite from DENV-infected mosquito	^27^
2014	South Korea	Technician	The staff member worked in a BSL- 2 facility, infecting mosquito cells with DENV-2. While transferring the virus solution from a tube to a syringe for filtration, she accidentally pricked herself with the needle. Initially, the needle cap was on, but as she continued working, she removed it to suction the remaining solution. She sustained the injury while trying to recap the needle.	Needlestick injury	^24^
2018	United States	Researcher	The case-patient worked with DENV-4 virus for 2 weeks before falling ill. They often experienced splashes during work but did not change gloves. Handwashing after glove removal was inconsistent. They sustained a wound on their left ring finger, potentially contaminated by the virus. In addition, they mentioned the risk of touching mucosal surfaces with their lab coat sleeve during work.	Possible DENV aerosol or parenteral exposure	^22^

#### Disinfection and decontamination

DENV inactivation has been studied using various methods and conditions, which are summarized in Table 5. Heat inactivation at 56°C for 30 min and 70°C and 121°C (autoclaving) for 15 min ensured complete inactivation of all DENV serotypes.^50^ Similarly, ultraviolet (UV) inactivation is highly effective, with 45 min of exposure leading to the absence of a viable virus.^50^ Chemical inactivation methods have shown robust results, such as using 0.05% formalin at 2°C to eliminate viral activity^51^ or applying a mixture of 1% tri-n-butyl phosphate (TnBP) and 1% Triton X-45 at 31°C,^52^ which inactivates DENV-1 in plasma and cryoprecipitate within 10 min. The C3 isomer, at a concentration of 10 mM, inhibits DENV-2 replication under illumination or in darkness, with complete suppression observed at 40 mM concentrations.^53^

Photodynamic inactivation methods leverage compounds such as methylene blue (MB), with results showing complete DENV inactivation at concentrations of 1.0 µg/mL when applied at a distance of 2.5 m within 5 min. Higher distances or lower concentrations necessitate longer exposure times. The Mirasol PRT system reduces viral infectivity ranging from 1.28 to 1.81 log across different serotypes.^54^ However, it does not reach the detection limit, whereas the THERAFLEX MB-Plasma system achieves a ≥4.46-log reduction for all serotypes.^55^ UV-C exposure at standard doses (0.2 J/cm^2^) results in significant viral inactivation, exceeding 4.43–6.34 log reductions across DENV serotypes, chikungunya virus, and Ross River virus. Other studies demonstrated that no detectable DENV remains after exposure to aminomethyl-trioxsalen-containing supernatant under UV-A light (200 μW/cm^2^ for 10 min) or following UV inactivation with Isopropyl Naphthalene Acetate (INA) compounds.

## Discussion

### Divergence in Risk Group Classification

The international classification of the DENV presents a significant gap in the consistency of its risk assessment and categorization. Although RG2 suggests a moderate risk to individuals and the community, requiring standard laboratory precautions, RG3 indicates a higher level of risk, necessitating more stringent containment and control measures to protect both laboratory personnel and the public. Such discrepancies can lead to inconsistencies in research protocols, public health responses, and international collaborations. Differing classifications may affect the level of biosecurity measures implemented in laboratories, influencing the outcomes of research studies. In addition, the public health policies and strategies for managing dengue outbreaks could vary significantly between regions, potentially impacting the effectiveness of disease control efforts. Therefore, collaborative efforts between international health organizations and regulatory bodies are of utmost importance to reconcile these differences and establish a unified framework for the classification of the DENV.

### Inconsistent Recommendations for Risk-Based Biosafety and Containment Levels

Although the current biosafety and biosecurity regulations for handling the DENV are generally comprehensive, we suggest several areas of improvement to ensure that appropriate safety procedures are consistently applied across various laboratory operations and environments. One critical aspect is the precise criteria for when BSL-2 versus BSL-3 containment should be implemented, which sometimes can be ambiguous. For instance, routine diagnostic work with clinical specimens typically can be performed in BSL-2. However, activities such as the large-scale production of viral stocks and research involving the generation of aerosols may require the more stringent controls of BSL-3. In addition, it is essential to standardize biosafety regulations across regions and countries. Differences in biosafety regulations and standards between countries and organizations can lead to inconsistencies in implementation. By harmonizing these standards, particularly regarding PPE requirements and laboratory practices, a more cohesive and practical approach to biosafety can be achieved.

### Causes of Laboratory-Acquired Infections

Laboratory-acquired infections present significant challenges to occupational health and biosafety, with human factors consistently emerging as a critical contributor to such incidents.^56^ Analysis of DENV LAI cases demonstrate that lapses in adherence to safety protocols, inadequate training, and lack of situational awareness are recurrent causes. Examples include improper handling of infectious samples, inconsistent use of PPE, needle-stick injuries, and exposure to aerosolized pathogens. Working with dengue-infected mosquitoes introduces additional risks, including potential bites from escaped or improperly secured mosquitoes, as well as accidental exposure during the handling of infected colonies. These incidents underscore recurring gaps in adherence to biosafety protocols, inconsistent training, and inadequate containment infrastructure, even in facilities designated for work with infectious agents. Addressing these risks requires implementing physical containment measures, such as double-screened entrances, secure insectary design, and strict mosquito-handling protocols, alongside PPE, such as long-sleeved clothing and gloves. As well as DENV-specific interventions, it is recommended that general comprehensive biosafety training be tailored to specific laboratory risks, including regular refresher courses and practical simulations of emergency scenarios. Moreover, promoting a culture of accountability and awareness, where personnel feel empowered to report near-misses and hazards, can significantly reduce risks. Integrating behavior-focused training, enhanced communication strategies to reinforce the importance of standard operating procedures, and standardized protocols is critical to minimizing risks associated with laboratory-acquired DENV infections and ensuring a safer working environment.

### DENV Infectious Dose

A critical gap in knowledge is highlighted by the lack of evidence on the infectious dose of DENV across different hosts and vectors. This gap complicates accurate risk assessments, limiting public health strategies and designing effective interventions. Understanding the exact infectious dose needed to infect mosquitoes is crucial for modeling outbreak dynamics and evaluating the efficacy of vector control measures. Furthermore, clearer insights into the infectious dose for humans could improve guidelines for clinical management and containment strategies during outbreaks. Addressing this gap requires concerted research efforts to standardize the measurement and reporting of infectious doses, including using consistent units and methodologies across studies. This standardization would facilitate more accurate comparisons and data integration, leading to a better understanding of DENV transmission dynamics and ultimately aiding in developing more effective control and prevention strategies.

### Inactivation

There is sufficient validated information regarding the chemical, thermal, UV, and photodynamic inactivation, such as that presented in Table 6. However, there remains a gap in the absolute validation of common chemical disinfectants such as sodium hypochlorite and ethanol and commercial disinfectants such as Virkon. Information such as effective contact times and concentration would aid in effectively implementing these common disinfectants.

**Table 6.** Studies that describe dengue virus inactivation methods

Inactivation method	Evidence	Reference
Heat	“In the heat-stability evaluation, all serotypes were subjected to heat treatment at 56°C, 70°C and 121°C either in a wet or pressurised settings. As shown in Table 1, two out of six wells containing Vero cells monolayer of DENV1 and 3, three out of six wells of DENV2, and 4 out of six wells of DENV4 showed CPE under the treatment of 56°C for 15 minutes, indicating the viruses could tolerate these conditions.”	^50^
UV	“To determine the efficacy of UV treatment in inactivating DENV contaminated surfaces, viruses were spread on 6-well plate and exposed to UV light at varied times at room temperature. The results showed that all serotypes were completely inactivated after 45 minutes UV exposure or longer as shown in Table 2. It should be noted that under 15 minutes UV exposure, three out of six wells were CPE-positive with DENV1, five out of six wells were CPE-positive with DENV2 and 4, and four out of six wells were CPE-positive with DENV3.”	^50^
UV	“At the standard ultraviolet C (UVC) dose (0.2J/cm^2^), viral inactivation of at least 4.43, 6.34, and 5.13 log or more, was observed for DENV 1-4, CHIKV, and RRV, respectively. A dose dependency in viral inactivation was observed with increasing UVC doses.”	^55^
Chemical	“The purified virus was inactivated with 0.05% formalin at 2°C.”	^51^
Chemical	“DENV-1 was strongly inactivated in plasma and cryoprecipitate, respectively, within 10 min of 1% TnBP/1% Triton X-45 treatment at 31°C.”	^52^
Chemical	“The C3 isomer at a concentration of 10 mM inhibited dengue-2 virus replication when illuminated by a 20-W white light bulb for 1 h (Fig. 1A; illuminated). The C3 isomer can also completely suppress viral replication in the absence of illumination (Fig. 1A; unilluminated) or in total darkness (Fig. 1A; dark), but at a concentration of 40 mM.”	^53^
Photodynamic	“The culture of A, B, C preinactivation, and D noninactivated samples showed the presence of replicative viruses during the five successive passages. In contrast, no replicative virus was detected in inactivated samples, even after five passages.”	^57^
Photodynamic	“Dengue virus could be completely inactivated at 2.5 m in 5 min when MB ⩾ 1.0 μg/mL. However, when the distance reached 3.0 m, only greater concentrations of MB (2.0 μg/mL) could completely inactivate virus in a reasonably short time (20 min), and smaller concentrations of MB (1.0 μg/mL) could only completely inactivate virus using longer times (25 min).”	^58^
Photodynamic	“Platelet Treatment with the Mirasol PRT system resulted in an average of 1.28 log_10_ reduction in DENV-1 infectivity (Table 2). For DENV-2, the average level of viral inactivation was 1.45 log_10_, while for DENV-3, infectivity was reduced by 1.71 log_10_ reduction (Table 2). The greatest amount of inactivation was observed for DENV-4, with 1.81 log_10_ reduction (Table 2). Inactivation to the limit of detection of the assay system used was not achieved.”	^54^
Photodynamic	“For Units 1 to 3, the mean +- SD DENV infectious titer was 6.61 +- 0.19 log TCID_50_/mL in preinactivation samples (UT0) and 5.46 +- 0.2 log TCID_50_/mL in noninactivated samples (UT20h; Table 1). Immediately after inactivation, no replicative DENV was detected in any of Units 1 to 3 postinactivation samples (TT20h).”	^59^
Photodynamic	“Treatment of plasma with the THERAFLEX MB-Plasma system resulted in at least a 4.46-log reduction in all DENV serotypes and CHIKV infectious virus. The residual infectivity for each was at the detection limit of the assay used at 60 J/cm^2^, with dose dependency also observed.”	^60^
Photodynamic	“No detectable PFU was noted following 10 min of exposure of the AMT-containing DENV-1 supernatant to 200 μW/cm^2^ of UV-A or following 5 min of exposure to 1,000 μW/cm^2^.”	^61^
Photodynamic	“50 μM INA and 2 mM glutathione to UV radiation (∼365 nm, 5 min, ∼145 mW/cm^2^)” “10 μg/mL (35 µM) aminomethyl-trioxsalen (Sigma-Aldrich) (AMT) to UV radiation (∼365 nm, 2 min, ∼145 mW/cm^2^)” “Results (Table 1) showed that no plaques could be detected after inactivation with INA (3 min), AMT (5 min) or formaldehyde (5 d).”	^62^
Photodynamic	“No replicating infectious DENV was seen in post-treatment samples even after five rounds of propagation in C6/36 cells. Also, no replicating infectious DENV was detected in posttreatment samples in Vero cells.”	^63^

CPE, Cytopathic effect; DENV, Dengue virus; TNBP, Tri-n-butyl phosphate; MB, Methylene Blue; CHIKV, Chikungunya virus; RRV, Ross River virus; PFU, Plaque forming unit; SD, Standard deviation; TCID50, 50% Tissue culture infectious doses; AMT, 4′-aminomethyltrioxsalen hydrochloride; INA, Isopropyl Naphthalene Acetate; UT0, Untreated samples at time 0 (preinactivation); UT20h, Untreated samples after 20 hours; TT20h, Treated (inactivated) samples after 20 hours.

### Related Flaviviruses

Similar inconsistencies in biosafety and biosecurity measures are observed with other members of the Flavivirus family, such as the Japanese encephalitis virus (JEV), Zika virus (ZIKV), and West Nile virus (WNV). These pathogens share common characteristics, including their single-stranded RNA genome and transmission primarily through mosquito vectors, yet there are significant discrepancies in their global risk group classification and containment recommendations. For instance, JEV is classified as an RG3 pathogen in many countries,^64,65^ requiring BSL-3 containment for laboratory work, but specific guidelines still allow for work under BSL-2 conditions under specific circumstances. Similarly, the ZIKV, despite its association with severe congenital abnormalities and neurological complications, is inconsistently classified between RG 2 and 3 depending on national guidelines,^66^ complicating international research collaborations and containment strategies. The WNV, primarily classified as RG 3 in most regions,^67^ presents challenges due to its zoonotic potential and sporadic outbreaks, which require enhanced biosafety measures during diagnostic or vector competency studies. Addressing these disparities by developing unified biosafety protocols across the Flavivirus family would facilitate consistent risk assessments and improve global preparedness and response to emerging or re-emerging flavivirus threats.

## Conclusion

The investigation into biosafety and biosecurity practices for the DENV reveals significant knowledge gaps and inconsistencies. These deficiencies are particularly evident in the risk classification of DENV, infectious dose, and proper biocontainment measures. Developing and implementing standardized, evidence-based protocols and extensive investigation and assessment across all relevant sectors is imperative to address these challenges effectively. By closing these knowledge gaps and establishing robust biosafety and biosecurity frameworks, we can better protect public health and mitigate the spread of DENV.

## Data Availability

There is no specific dataset related to this study.
